# Novel Left-Sided Thoracoscopic Approach to Recurrent Tracheoesophageal Fistula and Post-Fistula Tracheal Diverticula

**DOI:** 10.3390/jcm12237251

**Published:** 2023-11-23

**Authors:** Dariusz Patkowski, Krystian Toczewski, Ergun Ergun

**Affiliations:** 1 Clinical Department of Pediatric Surgery and Urology, Wrocław Medical University, Borowska 213, 50-556 Wrocław, Poland; dariusz.patkowski@umw.edu.pl; 2 Department of Pediatric Surgery, Faculty of Medicine, Ankara University, AÜ Tıp Fakültesi Hst. No: 6, Ankara 06620, Turkey

**Keywords:** recurrent tracheoesophageal fistula, tracheoesophageal fistula, tracheal diverticulum, esophageal atresia, thoracoscopy, tracheoesophageal surgery

## Abstract

Background: Recurrent tracheoesophageal fistula (RTEF) is usually a consequence of leakage or other complications after esophageal atresia repair performed through right-sided access. This results in extensive intrapleural adhesions, and open redo surgery poses a challenge. Alternatively, endoscopic endotracheal fistula obliteration usually requires repetitive procedures, and its success rate varies significantly between centers. We present a novel approach to recurrent fistulas. The innovation is in reaching the fistula through the virgin field via left-sided three-port thoracoscopy instead of classical right-sided thoracotomy. Methods: This is a presentation of a new operative technique based on a retrospective case series of patients operated on at our department between 2016 and 2023. Results: Eight patients after esophageal atresia repair (six with RTEF and two with post-fistula tracheal diverticula) were successfully treated with left-sided thoracoscopy. There were no conversions. One patient required rethoracoscopy for chylothorax. Another one, after RTEF closure, underwent multiple endoscopic obliterations of subsequent tracheal diverticulum. No other major complications nor re-recurrences were noted. Conclusions: Left-sided thoracoscopy in redo esophageal atresia has the advantage of a “virgin” operative field and grants feasible access to the RTEF or tracheal diverticulum. We believe that this approach is worth further exploration because it combines minimal invasiveness with high effectiveness without all the consequences of a thoracotomy.

## 1. Introduction

One of the most common reasons for reoperation after esophageal atresia repair is undiagnosed upper tracheoesophageal fistula or recurrent tracheoesophageal fistulas (RTEF), occurring in 1.9–16% of cases [1,2,3,4,5]. Although RTEF is rare, it is also one of the most complex and serious late complications of esophageal atresia surgery. Not only fistulas but also tracheal diverticula (TD) as a remnant of the closed fistula may be seen. RTEF is usually a consequence of complications, mainly leakage, after primary right-sided repair of esophageal atresia [2,3,4]. It results in extensive adhesions and scarring developing in the surrounding tissues and pleural cavity. Any reoperation through the right side is therefore challenging.

Current treatment is endoscopic obliteration of the fistula or open right-sided surgical removal of the fistula or diverticulum. The reoperation is either open or endoscopic, the recanalization rate is still over 20%, and the mortality is up to 10% [2,3,4,6,7,8,9]. 

We found that in all our cases, the RTEF/diverticulum was located to the left of the esophagus and was accessible through the left side. This study aims to present a novel left-sided thoracoscopic approach for RTEF and TD as a complication of EA surgery. 

## 2. Materials and Methods

The study was approved by the Bioethical Committee of Wroclaw Medical University (KB–18/2020). The research has been performed in accordance with the Declaration of Helsinki, and informed consent for retrospective review was obtained from all participants’ legal guardians.

We retrospectively reviewed the medical records of patients diagnosed with RTEF or TD who underwent left-sided thoracoscopic repair since the introduction of the method in 2016. We also analyzed the operation video recordings and completed the data from the outpatient clinic and telephone consultations. We define major complications as grade IIIa and higher according to the Clavien–Dindo classification [10].

### Thoracoscopic Treatment and Patient Management

**Figure 1:**
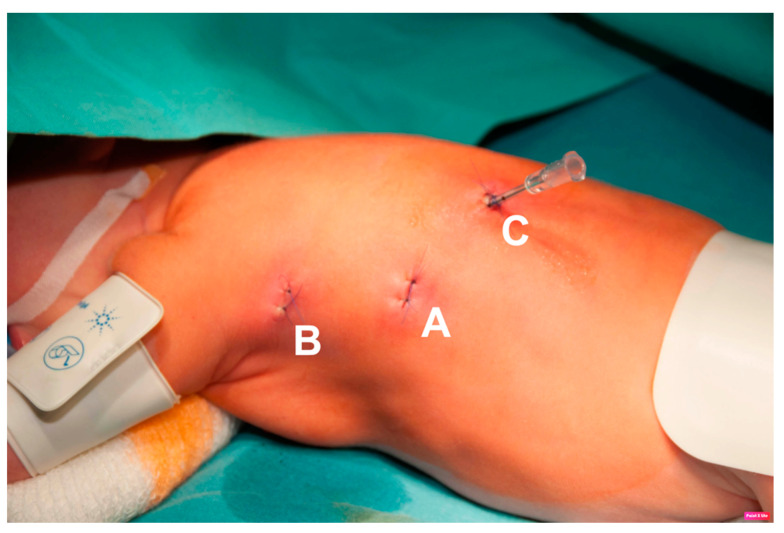
Trocar placement. A—5 mm optical trocar, B and C—3 mm working ports. This is picture of one of the first cases; now the position is prone.

**Figure 2:**
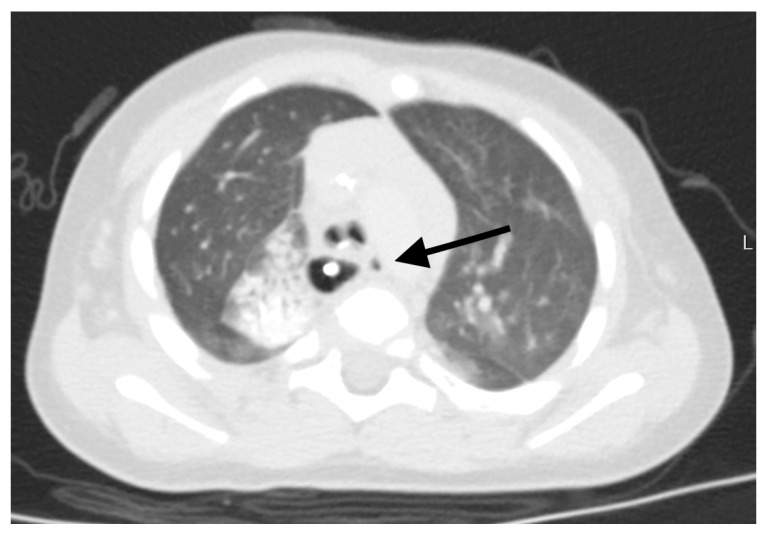
Massive inflammatory changes and suspected adhesions to the right from the esophagus. The arrow shows the RTEF with a diverticulum. Please notice its left position to the trachea and esophagus.

**Figure 3:**
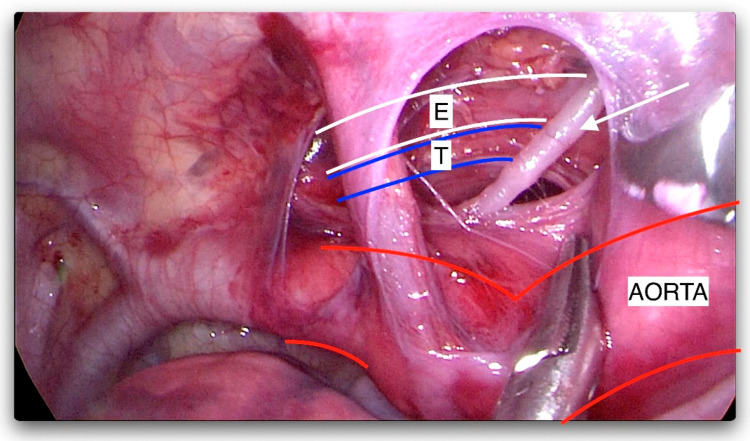
The mediastinal pleura opened; the thoracic duct dissected (arrowhead). E—esophagus, T—trachea.

**Figure 4:**
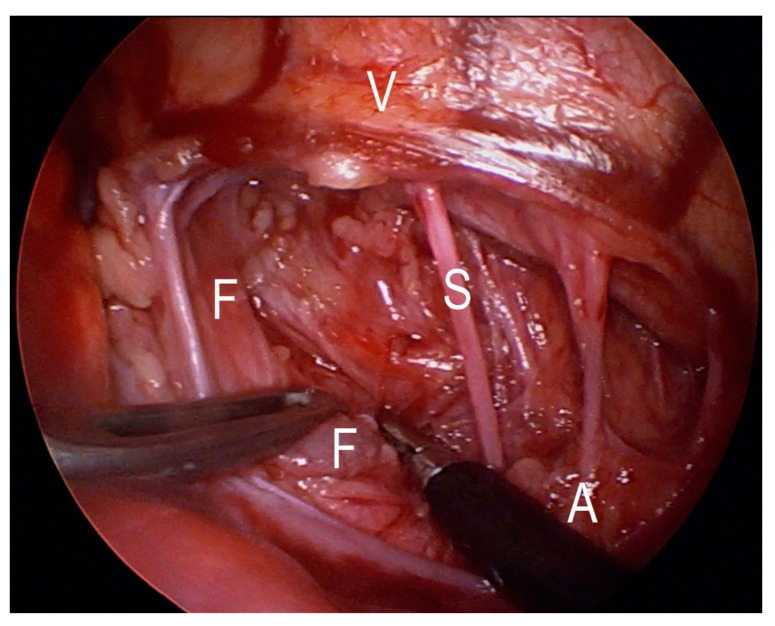
Deep dissection plane with spared aortal segmental vessels. V—vertebra, A—aorta, S—segmental vessels, F—fistula.

**Figure 5:**
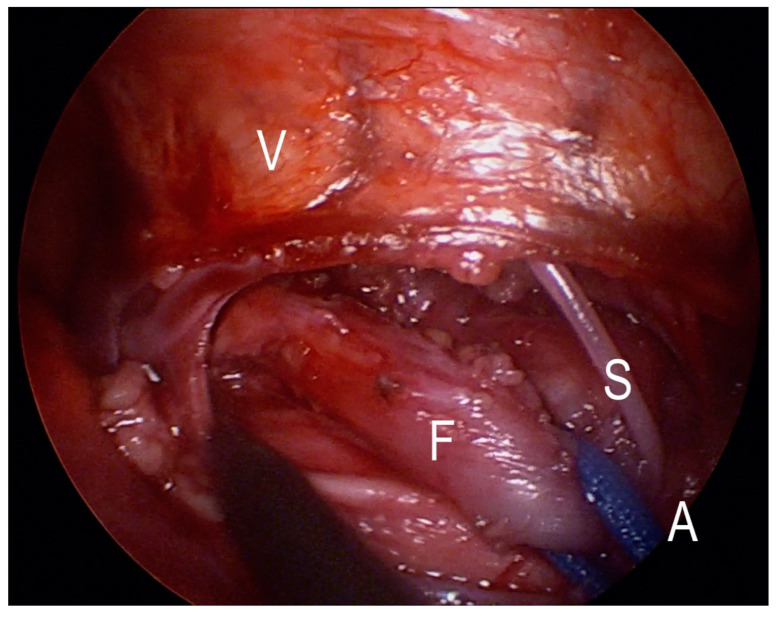
Dissected fistula on blue loop. V—vertebra, A—aorta, S—segmental vessels, F—fistula.

**Figure 6:**
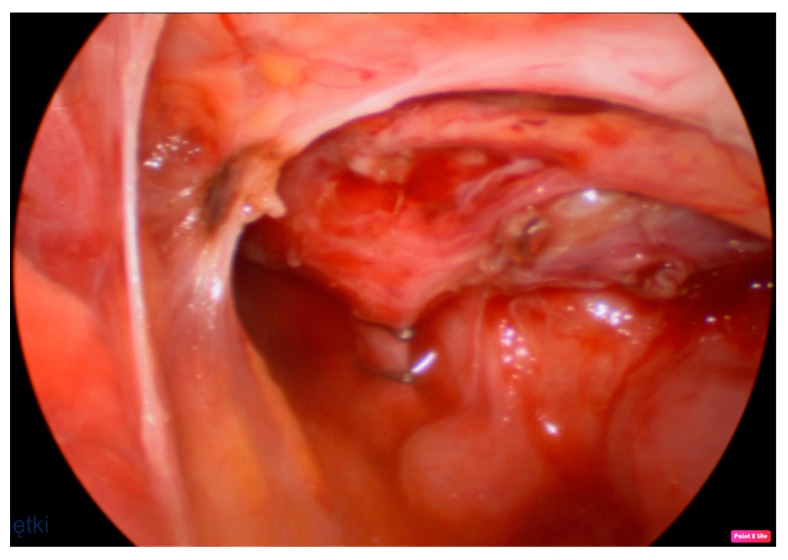
An example of fistula clipped on both sides.

**Figure 7:**
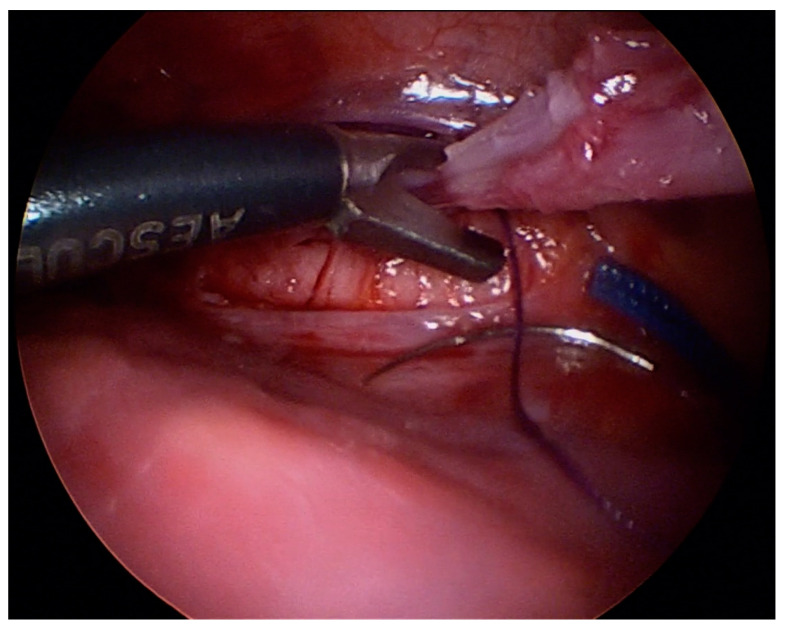
Transection of the fistula.

The first author, who operated on all the cases, has the experience of more than 350 thoracoscopic procedures for EA/TEF pathology. The fistula or TD position was precisely defined using a computed tomography (CT) scan to the corresponding vertebra. The operative technique was similar in all cases. All procedures started with rigid bronchoscopy to confirm the diagnosis, rule out other airway pathologies, and in later cases, to place a guide wire through the fistula to help its identification during thoracoscopy. Then, left-sided three-port thoracoscopy was performed in a prone position (in the first two cases, left side was slightly elevated). First, the optical 5 mm trocar was inserted in the next intercostal space below the tip of the scapula at the posterior axillary line (A in Figure 1). Pneumothorax with CO_2_ at 6 mmHg was obtained (flow 1–3 L/min). Then, the remaining two 3 mm working trocars were placed under direct view around the scapula above and below the optical port (B and C in Figure 1). The mediastinal pleura was opened according to preoperative fistula localization, based on the CT scan and tracheoscopy, usually between Th4 and Th6 (Figure 2). The aorta was bluntly dissected above and posteriorly to the arch, and its segmental arteries to the vertebrae were spared. The plane of the dissection went deep here, leading to identification of the trachea and the esophagus (Figure 3). The fistula was localized and dissected (Figure 4 and Figure 5). The method of fistula closure depended on intraoperative findings and the anatomy. Both ends of the fistula were either closed with ligation (Vicryl 3-0), transfixing (Vicryl 3-0), or titanium 5 mm clips (B. Braun Aesculap AG, Tuttlingen, Germany) (Figure 6). In some cases, all these methods were combined. For the clip applier, one of the 3 mm trocars was replaced by a 5 mm trocar. If feasible, the fistula was transected (Figure 7). For the tracheal stump, the technique was the same except that the esophageal end did not need closure. A chest drain was placed through the lowest trocar wound in all cases (as seen at Figure 1).

## 3. Results

Although patients were diagnosed at different ages, they all had common symptoms: recurrent respiratory infections. Three of four patients diagnosed before two years of age had also been choking during feeding. Diagnosis was based on symptoms and radiological examination, including esophagography or CT, and was always confirmed with bronchoscopy before qualifying for operative treatment. Three patients with RTEF had undergone unsuccessful attempts of endoscopic treatment with trichloroacetic acid (one, two, and four procedures accordingly) before surgery.

In the study period, we have had six cases of recurrent tracheoesophageal fistulas (one of them was probably a missed upper TEF on the primary surgery) and two symptomatic tracheal diverticula (chronic respiratory infections and cough). Six of them were referred patients primarily operated on in other centers, while two patients with TD were our cases. Patient’s general data are in Table 1.

All patients except one had unfavorable courses after the primary esophageal atresia repair with complications such as anastomotic leakage or pneumothorax. Patient No. 1, who started the series and pushed us to try the new technique, had monthly anastomotic leakage and a history of several right chest tube insertions. As a result, RTEF was found during reoperation. Patient No. 5 underwent reoperation twice in the neonatal period because of anastomosis dehiscence. Seven out of eight patients required dilations for esophageal stenosis before diagnosis. 

All patients were treated according to the operative technique described in the Methods section (left-sided thoracoscopy). There were no conversions to open surgery. The mean operative time was 177 min (130–255 min).

In two cases, we left the fistulas transfixed and ligated or clipped because they were too short for transection. In another case, after closing the tracheal end of the fistula, it was easier to transect it first and then suture at the esophageal end.

**Figure 8:**
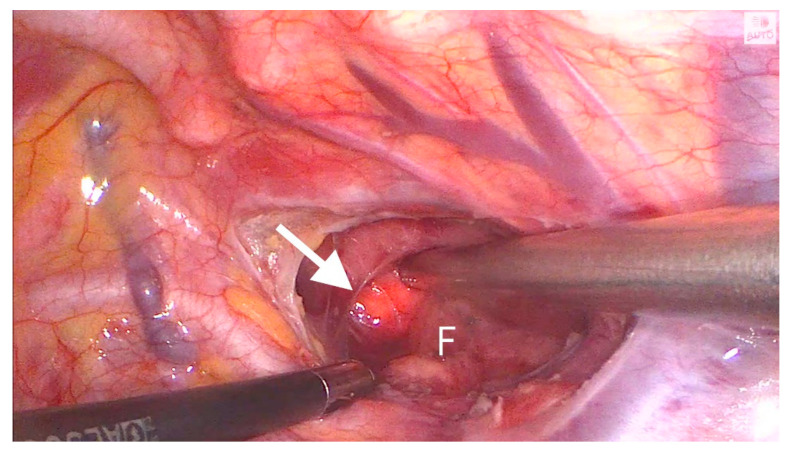
The endoscope light (arrowhead) close to the fistula (F) is visible.

In three cases, we had difficulties identifying the fistula. Using intraoperative esophagoscopy or bronchoscopy, the fistula was located and illuminated with the light of the endoscope, which allowed us to confirm the fistula position from a thoracoscopic view (Figure 8). Therefore, if feasible in subsequent cases, we placed a guide wire through the TEF during preoperative bronchoscopy and pulled it out from the esophagus with an endoscope, so the wire created a loop with both ends outside.

### Postop

The mean hospital stay postoperatively was 10 days (3–15 days). It is worth noting that last two patients went home 3 days after surgery. The complications rate was 2/8 (25%).

One patient (No. 5) developed significant chylothorax (up to 1100 mL/24 h). Conservative treatment (i.e., no enteral feeding, total parenteral nutrition, octreotide, drainage) was completely ineffective, so the patient was reoperated on six days after the TEF closure via left-sided thoracoscopy through the same wounds. As there was no identifiable source of chyle leakage, the previously opened mediastinal pleura was sutured, and the thoracic duct was ligated near the vertebrae. After the reoperation, the chylothorax diminished and resolved after four days.

Another patient (No. 6) had a persistent cough after the RTEF closure, and a postoperative endoscopy revealed a tracheal diverticulum, probably a result of insufficient dissection in difficult anatomical conditions. Therefore, over the next months, he underwent eight bronchoscopies with trichloroacetic acid obliteration of the TD. The TD diminished significantly, and symptoms resolved. The mean follow-up was four years (2 months–6 years), and there was still no recurrence. The first three patients underwent a postoperative endoscopy for recurrent respiratory infections, but no re-recurrence was found. Patients No. 4, 5, and 7 remained asymptomatic. In patients operated on for tracheal diverticulum, significant symptoms relief was achieved. The first patient who had the RTEF only transfixed and ligated without transection is currently over 3 years after the procedure and does not have any symptoms of re-recurrence of the RTEF. The second case without RTEF transection (only titanium clips) is the newest patient and remains under careful surveillance.

## 4. Discussion

According to the literature, the risk of RTEF is higher in children who have anastomotic leakage after primary surgery [8]. All but one of our patients had complications after initial esophageal atresia repair; thus our results support this idea. 

The idea of left-sided thoracoscopy arose when we faced a 7-month-old girl with an RTEF after almost one month of anastomotic leakage, and a CT and esophagogram not only confirmed the diagnosis but also showed extensive inflammatory changes, suggesting severe adhesions in the right pleural cavity with the fistula located to the left of the esophagus. As RTEF is usually a result of post-anastomotic leakage, which causes massive inflammation and adhesions in a pleural cavity, reoperation is technically challenging. Access to the diverticulum or RTEF through the left pleura seems to be easier because of the lack of postoperative adhesions and more direct visualization of the pathological structure, as the RTEF and diverticula in our experience appear to be mainly located on the left side of the esophagus.

The most challenging aspect of the operation is the identification and dissection of the fistula. In three cases in our series, we used intraoperative esophagoscopy and/or fiber tracheoscopy to illuminate the site of the TEF. Another solution is preoperative cannulation of the fistula with guide wire, as was performed in the last cases. We have already been utilizing it in a few cases of congenital H-type TEF, and it greatly facilitates intraoperative localization [11].

The proximity of the left thoracic duct passing near the aorta and the fact that it is not always identifiable poses an actual risk of injury of the duct, which justifies leaving the drain after surgery. Chylothorax is a potential complication of the left-sided approach as happened in one case, but it is manageable.

Thoracoscopy is becoming the gold standard for esophageal atresia and TEF in many centers [11,12,13,14], but RTEF surgery is still performed open [2,3,4,7,8,15]. The “classical” right-sided MIS approach for RTEF was mentioned only once in the literature by Rothenberg [16]. Left-sided thoracoscopy has already been used for primary repair in patients with EA and right aortic arch [17]. The left-sided thoracoscopic approach is innovative for recurrent TEF or tracheal diverticulum, although it has recently been described by Svetanoff et al. for the Foker procedure, mainly in LGEA, but also as a part of salvage strategy after failed primary anastomosis [18].

Alternatives are open or endoscopic treatment. The disadvantages of thoracotomy are already widely described when discussing esophageal atresia, and they include longer time to extubation and first oral feeding, longer postoperative stay, and higher risk of chest deformities [13,19,20]. Authors of the biggest series of open RTEF repair do not provide operative times or postoperative hospital stays [2,3,4,7,8]. Nevertheless, our last two patients went home 3 days after surgery which can be considered a fast recovery given the complexity of the reoperation and a scarred surgical field through the right chest. For the same reason, we cannot compare our operative times, but our mean 177 min for such a major surgery seems to be acceptable.

In a systematic review from 2013, Aworanti and Awadalla presented 108 patients treated with open surgery. The refistulation rate was 21%. In the recent literature, the re-recurrence rate is still 8–17%, with mortality up to 10% [2,7,9]. The complication rate in our series (25%) is comparable with these outcomes and comes with the advantage of minimal invasiveness.

When compared with endoscopic fistula obliteration, open surgery provides a higher success rate, requires fewer procedures, and gives fewer recanalizations [1,4]. On the other hand, endoscopic treatment is the least invasive. For endoscopy, the success rates vary significantly between studies from 37% to 100% in small series [1,21,22,23]. Although there are publications with encouraging results, in our presented cases, the endoscopic attempts failed. Compared to tracheal endoscopy, thoracoscopy seems to be more invasive, but nevertheless, also more precise. In our experience, thoracoscopic treatment is more successful. It can also be regarded as the potential second line of treatment in failed endoscopic closures or in patients who primarily do not qualify for endoscopic treatment because of the fistula size (too wide for obliteration) or accompanying diverticulum.

In the present study, the main outcomes were favorable: we had a low reoperation rate (one re-thoracoscopy for chylothorax and one case of endoscopic obliterations of subsequent TD), no fatal complications, and good follow-up, without re-recurrence of TEF.

The main limitation of the study is the small sample size. Although we are a referral center, we can only present this short series of eight cases as RTEF is very rare in our hands. These preliminary findings need to be confirmed in a larger group of patients to obtain more convincing results. Nevertheless, this is the only report of this technique thus far.

The technique itself is demanding and requires experience in thoracoscopy but combines all the advantages of minimally invasive surgery and a high success rate typical for open surgery.

## 5. Conclusions

Left-sided thoracoscopy in redo esophageal atresia has the advantage of a “virgin” operative field and grants feasible access to the RTEF or tracheal diverticulum. This approach is worth further exploration because it combines minimal invasiveness with high effectiveness without all the consequences of a thoracotomy.

## Figures and Tables

**Table 1:** General data of the patients.

Patient No.	Sex	EA Type	Primary repair	Referred	Diagnosis	Age at RTEF Diagnosis	Diagnostic Tests	Other Congenital Defects
1	f	distal TEF	thoracoscopic	+	RTEF	7 m	contrast, broncho	hypothyroidism, vertebral anomalies
2	m	distal TEF	thoracoscopic	−	TD	5 y	CT, broncho	PFO, PDA, pulmonary hypertension, laryngeal cleft
3	f	distal TEF	thoracoscopic	−	TD	23 m	broncho	PDA, ASD II, tracheomalacia
4	f	distal TEF	open	+	RTEF	5 m	CT, broncho	PDA
5	m	proximal and distal TEF	open	+	RTEF	5 y	CT, broncho	VUR
6	m	distal TEF	open	+	RTEF	23 m	CT, broncho	ARM
7	m	proximal and distal TEF	thoracoscopic	+	missed upper TEF	3 y	contrast, broncho	VACTERL
8	m	distal TEF	thoracoscopic	+	RTEF	8 y	CT, broncho	none

TEF—tracheoesophageal fistula, RTEF—recurrent tracheoesophageal fistula, contrast—esophagography, CT—computed tomography, broncho—bronchoscopy, PFO—persistent foramen ovale, PDA—persistent ductus arteriosus, ASD—atrial septum defect, VUR—vesicoureteral reflux, ARM—anorectal malformation, VACTERL—VACTERL association.

## Data Availability

Data is contained within the article.

## Abbreviations

RTEF	recurrent tracheoesophageal fistula
TD	tracheal diverticula
CT	computed tomography
